# Using a music microanalysis protocol to enhance instrumental practice

**DOI:** 10.3389/fpsyg.2024.1368074

**Published:** 2024-04-02

**Authors:** Guadalupe López-Íñiguez, Gary E. McPherson

**Affiliations:** ^1^Sibelius Academy, University of the Arts Helsinki, Helsinki, Finland; ^2^Melbourne Conservatorium of Music, The University of Melbourne, Parkville, VIC, Australia

**Keywords:** metacognition, microanalysis, musical development, motivation, self-regulated learning, musical practice, higher music education, professional education

## Abstract

The strategies that enable musicians to adapt their behaviors so that they can break through, feel energized, and perform well collectively distinguish what it is to be a self-regulated learner. These strategies range from one’s ability to monitor thoughts and actions to being able to navigate and control one’s emotions, especially when feeling frustrated or anxious. Given the challenges of the music profession, it becomes imperative for teachers to equip their students with the necessary skills to self-regulate their own actions, feelings, and thinking so that they are eventually able to cope with the demands required of a contemporary professional musical career. In this study, we focused on the self-regulatory engagement of four master’s level cellists who were enrolled in a prominent European higher music education institution. Our data comprised self-regulated learning-based diary-reports that describes the students’ practice of self-chosen, especially demanding passages as they prepared for a public recital. Results depict differences between the musicians according to the efficiency of their practice leading up to a formal public recital.

## 1 Introduction

Developing autonomy and control of one own’s practicing routines and learning strategies is a never-ending quest for performing musicians. A progressive and time-intense practicing process starts at an early age and becomes more important as a developing musician learns to take charge and self-regulate their behavior. Practice autonomy is crucial when musicians transition from higher education to the music profession—an ever-changing, artistically creative, and economically challenging arena. Under such circumstances, one might assume that music graduates would be prepared to step into the industry reality and take charge by themselves, especially in terms of knowing how to practice their instrument and being autonomous in their artistic decisions and strategic learning (McPherson, 2022; Zachariou and Bonneville-Roussy, 2024). An ideal scenario, given today’s imperatives of being a professional musician, would be that young musicians enter the profession sufficiently prepared to take on their roles in the vast number of possible employment scenarios within the music profession that are available to them without the ongoing need to rely on the support of a teacher or institution.

The centuries-long conservatoire tradition—based on the master-apprentice model—does not always help to build the required autonomy to learn new and old repertoire (Gaunt et al., 2021; Pozo et al., 2022). Instrumental and vocal students spend a large part of their lives listening to the advice and observing the playing of their teachers in one-on-one lessons—and that of experts in masterclasses or competitions. This includes explanations and demonstrations on *how* to practice, as well as modeling of the *in which* ways to practice. As a result, many music students remain dependent on their professional mentors’ feedback and advice till the end of (and sometimes beyond) their professional studies. Considering the crude neoliberal reality of modern-day performing music careers—where creativity, innovation and autonomy are key elements of flourishing—this pedagogical approach seems inadequate and inefficient (Evans and Ryan, 2022).

Given the challenges of the music profession, it becomes imperative for higher music education—and, generally, for liberal arts universities—to embrace the responsibility and leadership required to equip performing students with the necessary skills to flourish personally and professionally as well-rounded musicians (e.g., López-Íñiguez and Bennett, 2020; Palmer and Baker, 2021). For decades, educational social psychology research has acknowledged that one approach for achieving this includes fostering students’ metacognitive engagement with their learning by teaching them how to take charge of their learning (McPherson and Hattie, 2022; Pozo et al., 2022). In short, great teachers encourage their students to self-regulate their actions, feelings, and thinking by helping them become progressively more autonomous and independent so that they are eventually able to cope with the demands required of a contemporary professional musical career (McPherson and Hattie, 2022).

## 2 Theoretical framework

### 2.1 Self-regulated learning and professional music performance

Research shows that more proficient and resilient musicians have developed a range of organizational competencies and skills that they draw upon when preparing for a challenging performance (Pecen et al., 2018). The ‘toolkit’ of strategies that enable musicians to adapt their behaviors so that they can break through, feel energized, and perform well collectively distinguish what it is to be a self-regulated learner (McPherson, 2022). These strategies range from the ability to monitor one’s own thoughts (cognition) and actions (behavior) to being able to navigate and control your own emotions (affect), especially when feeling frustrated or anxious (McPherson and Zimmerman, 2011; McPherson, 2022). The process of triangulating these three basic and interrelated human capacities equips music professionals with the toolkit of skills needed for them to develop their own distinctive “learner identity” that will subsequently support them throughout their professional lives (e.g., López-Íñiguez and Bennett, 2021). As McPherson (2022) states, “becoming self-regulated in the mastery of music involves being able to recognize a challenge, understand the scope and nature of this challenge, focusing your motivation to deal with the challenges, enacting strategies and plans to overcome the challenge, and evaluating your progress toward overcoming the challenge” (p. 556).

The above processes are integral to the development of musical expertise (Zimmerman and Moylan, 2009; McPherson et al., 2017; McPherson, 2022). Indeed, in recent years, music research has attended to help graduate music students (e.g., Jabusch, 2016; Antonini Philippe et al., 2020; Rodríguez-Cortés and Casas-Mas, 2023) as well as professional musicians (e.g., López-Íñiguez and McPherson, 2020, 2021) to elicit their general preparation for performance with the aim of improving their self-regulatory skills. Drawing on work by Zimmerman (2002), these studies include efforts to adapt and refine existing self-regulation questionnaires and rubrics from general learning domains that serve diverse purposes in helping people to manage their learning, motivations, and emotions in a more agentic way. Another step forward in supporting learners of different ability to proactively engage with self-regulation, has been the development of a technique known as *microanalysis*, which supports learners in establishing and assessing specific learning goals and strategies to manage their learning in a variety of contexts (Cleary and Callan, 2018), including music (e.g., McPherson, 2022).

### 2.2 Microanalysis of self-regulated learning in music

**Figure 1:**
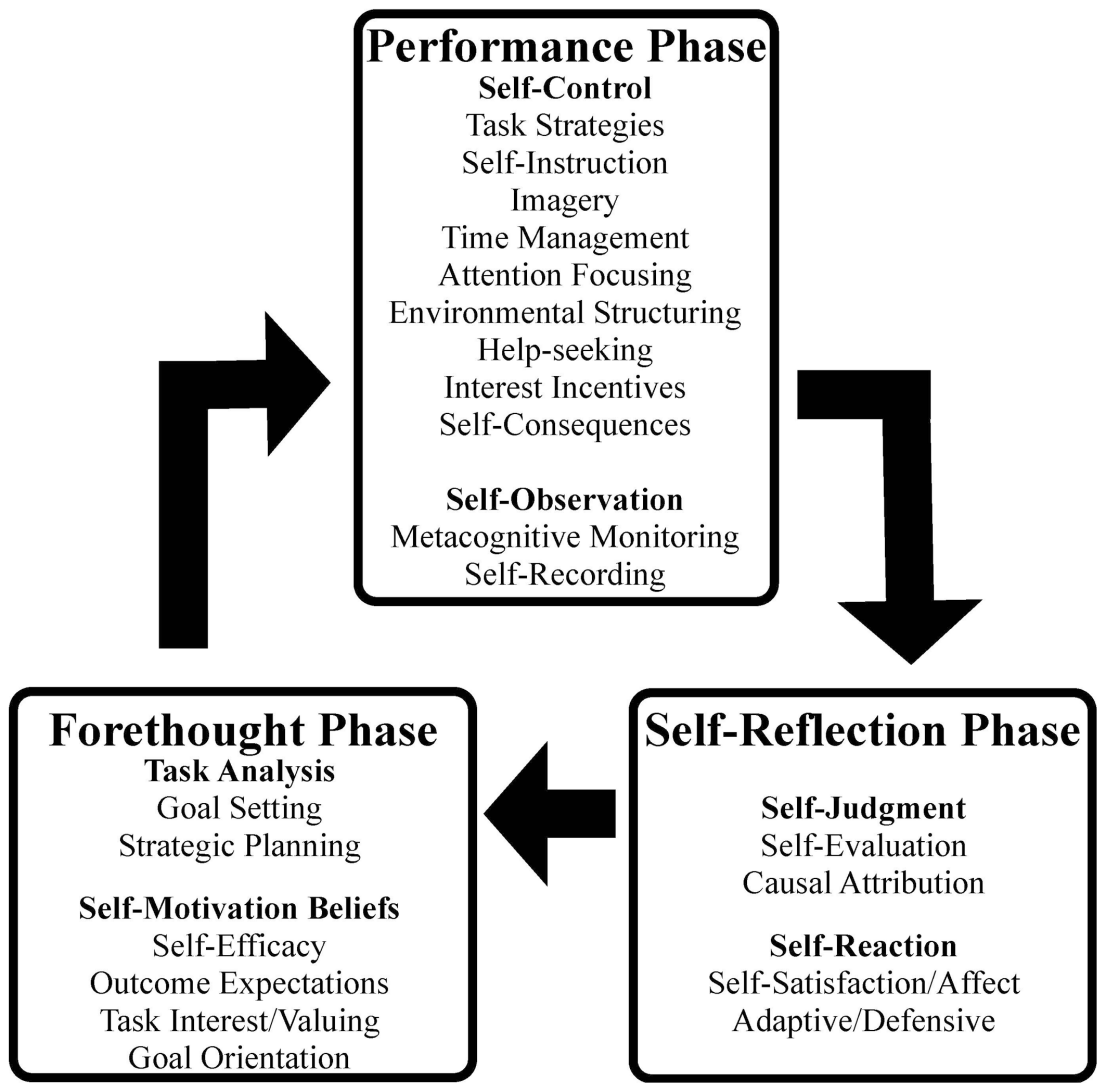
Phases and sub-processes of self-regulated learning in music. Reprinted with permission from McPherson ((2022), p. 555).

Self-regulated learning microanalysis is a helpful technique to examine how learners engage in authentic learning and performance activities according to the cyclical phases and sub-processes of the model depicted in Figure 1. This technique helps to assess how learners in any field of domain apply, monitor, and adapt strategies and actions to improve their own actions, cognition, and affect when learning anything in a specific moment (Cleary and Callan, 2018; in music, McPherson, 2022). The technique is structured by (1) Selecting a well-defined task; (2) Identifying target processes that need most attention; (3) Developing self-regulated learning microanalytic strategies that align to the specific self-regulated learning technique that is being targeted; (4) Linking cyclical phase processes (before, during, after) to task sub-process (behavior, cognition, affect); and (5) Evaluating and planning for the next attempt, practice session, or performance (Cleary et al., 2012).

Over the past decade, a music microanalysis technique has been developed by Author 2 and his colleagues (McPherson et al., 2015; Osborne et al., 2021) for use by musicians of various abilities. The “Optimal Music Practice Protocol” (OMMP) they developed is based on the three-phase model of self-regulated learning (i.e., forethought, performance, and self-reflection) shown in Figure 1, with the expressed aim of cuing “students to describe their actions and then reflect critically on the strategies they choose to improve their playing *in-situ*.” (McPherson et al., 2019, p. 19). Most of the research using the OMMP has focused on the practice habits of undergraduate piano performance majors who rely heavily on their teachers’ feedback to prepare challenging repertoire for their end of semester recital examinations. To date, there have been no research studies using the microanalysis technique with musicians during the transition stage from higher education to music careers when no teacher feedback is available to them, and when they are preparing to play profiled concerts rather than exam recitals.

## 3 Aims of the research

With the above in mind, this study focuses on the self-regulatory engagement of four highly skillful (i.e., invested in their studies, giving regular trial performances, having been accepted to a highly competitive study program) classical music performance students enrolled in a prominent European higher music education institution. We followed their self-directed practice across a semester-long process of preparations for their own virtuoso classical-romantic repertoire recitals at profiled concerts. Our data comprised self-regulated learning-based diary-reports that described the students’ practice of self-chosen “specially demanding” passages. The following questions guided our research:

- What sub-processes of self-regulated learning (as shown in Figure 1, and described in the OMMP), did the musicians use to evaluate the quality of their practice sessions, and how might these be different across the four musicians in relation to their own progress and their chosen repertoires? (i.e., non-comparative study).
- In what ways, if any, did the use of the OMMP self-regulated learning microanalysis technique help the musicians improve their performance of challenging passages of virtuoso repertoires when independently preparing for public concerts at profiled venues?

## 4 Method

### 4.1 Sample

Participants included four high ability performing cellists enrolled in official graduate programs at an elite European higher music education institution. At the time of data collection, all were combining the final year of their master’s degree in musical performance with remunerated professional performing activities. These participants—who were transitioning from higher music education to the music industry—were born in the early 1990s. All four musicians were completing a master’s degree in cello performance and had considerable performance experience in professional chamber and orchestral settings. They had also completed their undergraduate degrees and progressed directly to the master’s degree. To ensure maximum variety between them (Stake, 2005), the participants comprised two local and two international master’s students. To ensure the anonymity of participants, their names have been changed (see Table 1).

**Table 1:** Demographics of participants.

	Olga	Lukas	Xie	William
Birth year	1993	1991	1993	1990
Gender	Female	Male	Female	Male
Degree	Master’s	Master’s	Master’s	Master’s

### 4.2 Materials and procedure

This is a qualitative, purposeful multiple-case study (e.g., Yin, 2017) that involves a pedagogical intervention of self-regulation with four cellists. It is a non-comparative study that includes replications across the four cases to investigate the levels of sophistication of their use of self-regulated learning strategies across three practice sessions as they prepare unknown virtuoso repertoires for a profiled concert (see next section for details).

The participants were accessed via an open invitation to participate in the study, sent by the Head of the Department in charge, and they were offered study credits for their participation. To ensure this was a project-based learning to foster students’ autonomy, the participants agreed on the following aspects prior to the commencement of the study:

1. Choosing a recital repertoire that included pieces that were unfamiliar for them.
2. Reading sources connected to the repertoire that they thought could inform their artistic work (e.g., biographical, musicological).
3. Not bringing this repertoire to their regular cello lessons or to master classes.
4. Not listening to existing audio−/video-recordings of this repertoire.
5. Not attending live concerts where this repertoire was performed.

The reasons we chose to restrict the participants from listening to existing recordings of their chosen repertoires, or to prepare their recitals with other performing experts was twofold. First, in contrast to the practice of professional musicians, music students tend to rely more heavily on external regulators to inform their practice in the very early stages of learning (e.g., Volioti and Williamon, 2016); and second, when graduate musicians premiere contemporary repertoire, they typically do not have recordings, recent performances, or *status quo* performance advice available to them. Thus, the focus of the study was to delve into the students’ ability to self-regulate their preparation for their recital entirely by themselves, and to minimize any forms of external regulation that might impact on the choice of self-regulatory processes they employed for mastering the repertoire to be performed at a public event. We saw this as a first step for subsequently expanding the parameters of the current study for use with larger samples of students in which the microanalysis technique can be compared with other forms of practicing that include various forms of self and external regulation.

The study had three phases (see Table 2). In phase 1, the participants selected repertoire that was unfamiliar to them, based on an exhaustive list comprising all cello and piano sonatas from the classical to the early romantic periods. Participants were free to choose their own repertoires according to their interests. The reason for this was that we wanted the participants to be motivated and satisfied about their choices, and to be able to play the repertoire they selected in other contexts after the study, particularly given that the process involved many hours of dedicated practice during various stages across the semester. Furthermore, interest and motivation has been linked to higher self-regulation in musicians (e.g., Miksza, 2006; Evans, 2023). Regarding the selected repertoires, Liebmann (1806); Beethoven (1815); Smyth (1887); Martucci (1880) and Herzogenberg (1886). All participants then practiced on their own and with their pianists for 6 months, before giving a preparatory concert that was performed in front of the first author. This concert was videorecorded, and subsequently, the participants watched their own performances and were interviewed by the first author, to identify and select specific passages that they found challenging and that they would like to improve through their participation in this intervention.

**Table 2:** Phases of the study.

Phase 1	Phase 2	Phase 3
Selecting the repertoire. Pilot concert (videorecorded). Interview to select the most challenging passages.	3x study sessions (videorecorded). Applying the Optimal Music Practice Protocol *in-situ*.	Public concert (videorecorded). Interview to analyze the effectiveness of the intervention.

In phase 2, participants received an adapted version of the “Optimal Music Practice Protocol” (OMMP; McPherson et al., 2015, 2019; Osborne et al., 2021). The microanalysis protocol was administered across 3 stages of practice (before, during, after) in each practice session, where the participants focused exclusively on the challenging passages they had selected to master. The OMMP is useful for undertaking a self-analysis of what participants planned in preparation to their practice, what they did/thought/felt during the actual practice, and how they assess the practice session immediately after it had concluded as well as after watching a videotape of the practice. The OMMP booklet includes Likert scale items as well as open-ended questions and can be accessed via the link in the Supplementary material section of this article.

Within phase 2, musicians responded to the first section of the OMMP diary, that was completed before each practice session, and included questions about the tasks (i.e., goal setting, strategic planning) and self-motivation beliefs of these musicians (i.e., self-efficacy, outcome expectancies, task interest, and task value). The second section was carried out during their practice, where they reflected on issues of self-control (e.g., maintenance of concentration and interest, tactics used, structuring of environment) and self-observation (e.g., self-talk during problem solving, keeping record of progress). Finally, the participants provided responses to the third section, after watching a videorecording of their practice, where they made self-judgments on (1) the effectiveness of the practice, (2) their causal attributions for the quality of their practice, and (3) their affect and overall feeling of achievement.

In phase 3, participants played a public concert at a profiled venue, which was also video recorded. After the concert, the participants watched the recording and were interviewed to assess the effectivity of the intervention on the outcome of the challenging passages they had identified and worked on with the aid of the OMMP. All interviews were transcribed verbatim for data analysis.

### 4.3 Ethics

Ethical approvals were obtained prior to the study from the Research Ethics Committee at the university where the study took place. Students signed consent forms following the guidelines of the local Advisory Board on Research Integrity, and students were not obliged to participate.

### 4.4 Analysis

For this study, we applied the self-regulated learning microanalysis technique to analyze the learning strategies participants used before, during, and after each practice session, in connection to the challenging passages of classical-romantic repertoires, and whether they became more complex across sessions. We employed a self-regulation-grounded thematic analysis of the behavior, affect, and cognition the participants reported to improve their learning and performance of the selected passages, as well as their self-rating of improvement across sessions. During the deductive analysis, we read through the dataset multiple times, looking for meaningful themes (e.g., Braun and Clarke, 2012). As this is a multiple-case study, a summary of each individual case, from less to more sophisticated, is reported in the next section. In line with Yin (2017), this is followed by cross-case conclusions.

## 5 Findings

In the following sections we summarize how each of these musicians worked on the repertoire set for this study, using the OMMP to cue and guide their musical practice and preparation for their recitals. Each comment by the musicians includes a comment in brackets that identifies the self-regulated learning sub-processes shown in Figure 1, for which the comment aligns.

### 5.1 Olga

#### 5.1.1 Forethought phase

Olga chose the cello entrance of the first movement (Allegro) from Hélène Liebmann’s *Grande Sonata for Cello and Piano* (1806) and spent 10, then 17 and then 11 min for each of the three sessions working on this passage. She chose this section of the piece because she was not satisfied with the intonation in the “leap to B flat which should not have been that bad” and “the quality of the sound when there is a string crossing and a position change at the same time” (Strategic Planning). Olga also acknowledged that she “simply could not play [this passage] that well technically” as she was “not so well prepared” (Self-Efficacy). She recognized that it is important “not to rely on luck when my mind tells me I am going to fail” (Outcome Expectations). To solve these issues, Olga thought it would be a good idea to develop “a better contact of the bow hair on the strings” (Strategic Planning), however, she did “not know how to practice [the bow crossing] or how to find confidence in myself to do that” (Self-Efficacy). She also wanted to have “more fireworks and playing it in a more interesting way, to engage the audience more” when interpreting the music (Goal Setting).

Olga started each session by playing the passage through to “figure out what to do” and assess the “situation” (Goal Setting). Her goals before each practice session remained similar, with a strong focus on staying calm “to control everything that was happening” (Goal Orientation), especially being “bow-mindful” to anticipate bow squeaks in string crossing or “practicing slowly and being aware of the intonation by listening carefully” during left hand position shifts (Strategic Planning). Other aspects that she considered included a “lightness in the phrasing and a good sound blend when playing with the pianist” (Goal Orientation). Although she acknowledged that she had “not learnt any new strategies for working on this passage,” her confidence about her self-chosen goals and strategies (e.g., bow control, shifts in relation to intonation), as well as her competence to master them was strong (Self-Efficacy). The reasons for choosing these goals and strategies included achieving “a personal best, so that I’m not embarrassed and do not put others down, especially the pianist” (Goal Setting), as well as “avoiding forgetting what I have learnt” (Strategic Planning).

#### 5.1.2 Performance phase

While practicing during the first two sessions, Olga felt defenseless as she could not “improve or achieve anything anymore!” (Self-Consequences; Attention Focusing). She “hate[d] the feeling of recording myself as it made me shocked about the passage, whereas the pleasant music makes me feel neutral” (Self-Recording; Metacognitive Monitoring). Olga stressed however, the importance of “progressively normaliz[ing] the feelings about this passage and recording it” (Self-Recording), although in the last session, she felt “funny to succeed in front of the camera as I was just lucky, while disappointed and frustrated because I am not a perfect machine, obsessed with this [passage]” (Self-Monitoring). Olga also acknowledged that drinking coffee before practicing made her “extremely anxious” (Environmental Structuring). Overall, Olga thought “about new musical things in phrasing”; having “thoughts on improving little things through the influence of historically informed performance practice” (Help-Seeking). She consciously tried to develop a “technical control for bow squeaks, accentuation and articulation” (Self-Instruction), as well as improving the “intonation using double stops as cues [and being more aware of] the differences between B flat and sharp when comparing them to Baroque tunings” (Attention Focusing). She wanted to become “more fluent musically using direction in micro phrases, [adding] clear mordents and more ornaments for phrasing, and vibrato” (Task Strategies).

#### 5.1.3 Self-reflection phase

After each practicing session, Olga consistently reported an improvement in her concentration because “the [OMMP] tool is really useful in helping me to focus better” (Casual Attribution). She reported that this type of practice preparation and microanalysis had helped “especially this time in my life, when I have so little time and so much music to be performed” (Adaptive). Furthermore, she understood “how important this kind of practicing is and how it helps the final outcome; I did not understand it instantly I must say, but I definitely try to focus better from now on certain tasks” (Self-Evaluation; Adaptive). Although her sense of strategy effectivity and goal achievement after sessions was not strong, she had “not master the passage sufficiently” (Self-Evaluation), and she was initially reluctant to “work on the passage or think about it in terms of strategies” (Defensive). She did, however, acknowledge that being more aware of her goals and strategies helped in developing “more quality in the sound, intonation, and phrasing” (Self-Evaluation; Causal Attribution). During the actual concert, she reported “feeling a bit nervous, indifferent, and insecure” (Self-Evaluation), and although she “felt embarrassed [because] I nevertheless failed with some other passages” (Self-Evaluation), the passage she had practiced with the OMMP tool “came out the best; I was more courageous and had a better flow, and I was thinking, oh, it is better in tune than I thought” (Self-Satisfaction).

### 5.2 Lukas

#### 5.2.1 Forethought phase

Lukas chose bars 157–182 and 217 to the end of the last movement (Allegro Vivace) from Ludwig van Beethoven’s *Cello Sonata in C Major no.4, op. 102* (1815). He spent less than 10 min per session to work on these passages. Lukas identified a few blind spots he wanted to improve, acknowledging that many of them he did not realize during the pilot concert, but only after watching the video, which he considered “an eye-opening activity” (Task Interest/Valuing). He wanted to work on this passage because there were “sudden moments where we were not being completely together with the pianist” (Goal Setting) because “when you play them on your own, they are fine, but then in the concert everything is not like 100%” (Self-Efficacy; Goal Orientation). He also mentioned that “the intonation and the dynamics [of the selected passage] were not so spot on [even if these were the ones] I had worked most on, [but clearly] with not so successful strategies” (Strategic Planning).

For Lukas, his goals remained similar across the practice session, as he was consistently looking to “play the passage right while adjusting to the piano phrasing and the dynamics” (Goal Orientation). He also wanted to work on the “intonation in relation to position shifts and changes while being relaxed, for which he decided to make “rhythmic variations with 16th notes” and “playing around with the passage by using different positions and fingerings, sudden changes in sound quality” (Strategic Planning). To achieve these goals, Lukas’ strategies before practicing included “reading the score and visualizing it internally, focusing on its symbols” (Strategic Planning), using “[a long note played by] the tuning machine for guiding intonation while playing the passage slowly” (Strategic Planning), as well as focusing on “playing dynamics carefully and slowly, [working on] position changes through specific intervals and changing across strings more carefully [while playing at a] slow tempo [to assess] where is the weak spot in the shifts” (Strategic Planning). Lukas’ confidence in achieving and mastering these goals, as well as his personal interest and value in the longer term for carrying them out were rather high during the first two sessions, whereas they all dropped in his last practice, as he acknowledged experiencing a certain “lack of energy [and] pressure to work harder as the concert approaches, since I want to succeed” (Self-Efficacy). Like the external reasons that were given by Olga, Lukas explained that he engaged with these goals and strategies because he wanted to achieve his “personal best” (Outcome Expectations) and “[keep improving] while not letting the research, audience, and pianist down” (Outcome Expectations; Self-Efficacy).

#### 5.2.2 Performance phase

Regarding the actual practice sessions, Lukas was especially focused on feeling “the right mood, being focused on my goals and not [getting] too emotional” (Attention Focusing; Metacognitive Monitoring). Indeed, Lukas acknowledged that the longer he practiced, “the more frustrated I felt, [thus needing to] stay neutral and positive” (Self-Instruction). There were recurrent thoughts across the sessions, which included “feeling good to go on as all is going well” and wondering “if there was something that I did wrong, like the hands need to relax a bit more and be better coordinated for the quality of the passage” (Task Strategies, Self-Instruction). There were a variety of strategies he used to improve this passage, like “picking the G note with the tuner and playing the passage on top, to see if I am again wrong” (Task Strategies). He also made “rhythmic variations, being more and more progressive with tempo” (Task Strategies) and worked on “making dynamics clearer by listening to the contrast and thinking about it in relation to the piano balance, but also using bow speed as a new discovery to improve [the dynamic range]” (Task Strategies).

#### 5.2.3 Self-reflection phase

After each practicing session, Lukas mentioned that his concentration, sense of practice efficiency and effectivity of the strategies he chose were “quite good,” even if this feeling dropped slightly in the last session (Self-Satisfaction). He also mentioned that he “would not have used so much time and effort to this passage without this [OMMP tool] method, so I’d say it was very useful since I’m happy with the result” (Self-Satisfaction). The use of the microanalysis encouraged him before the concert, as he “felt determined and safe to go and play it, since I know how much time and effort, I have put to practicing this” (Self-Satisfaction). During the interview post-concert, Lukas said he was “happy with the end-result [as] I achieved 95% of what I was aiming at; I especially remember being able to play the dynamics out as well” (Self-Evaluation; Self-Satisfaction).

### 5.3 Xie

#### 5.3.1 Forethought phase

Xie chose the intermezzo of the 3rd movement (Andantino Falebile), and the scale and finale of the 4th movement (Allegro) from Giuseppe Martucci’s *Cello Sonata op. 52 in F-sharp minor* (1880), as well as the pizzicato of the 1st movement (Allegro Moderato) and the beginnings of the 2nd and 3rd movements (Adagio non troppo; Allegro Vivace e grazioso) from Ethel Smyth’s Cello Sonata no. 2, op. 5 in A minor (1887). To work on these passages, she spent 21, then 35 and then 77 min for each of the three sessions, without breaks. Xie explained that all these sections presented “intonation issues” during the pilot concert, as well as “issues of acoustic [as] the place where we were practicing with the pianist before the concert was different, so I need to change some articulations for acoustic reasons” (Strategic Planning). Xie also said that, generally, she had “interpretation shortcomings in these passages” (Outcome Expectations) and would like to get over her “worries and fears [and] give more in the performance while staying calmer physically; otherwise, my hands usually become too stiff and less flexible” (Outcome Expectations, Self-Efficacy). For the Martucci movements, she wanted to specifically work on character aspects and emotional connection with these movements by being “more playful, humorous, and fun, also in my communication with the pianist” (Goal Orientation); whereas for Smyth, she wanted to “connect emotionally with her as a woman composer at that time [since] I was reading about her, and she was lonely and challenged by many people” (Goal Orientation). Xie wanted to achieve her personal best, enjoy the learning process, and reduce the pressure of the concert deadline approaching (Strategic Planning).

Xie also wanted all passages to display “a sound I always have in my mind for colors and expression” (Goal Orientation, Outcome Expectations), for which she would “try several times to get closer to that using a different speed with the vibrato, and working on the bowing quality slowly, and watching the recording of my practice several times” (Strategic Planning). With Martucci in particular, Xie focused on “a big and sudden articulation shift, from staccato to a high position change, so you can be late or out of tune easily” (Strategic Planning), trying to “put all together, finding better intonation, playing it rather slowly, being aware of the left hand before the shifts, and then connecting this to the rest of both movements” (Strategic Planning). For Smyth, she tried to “bring the melody out a bit more, practicing only the upper line while singing internally while I play it softly and make a good phrasing connecting all notes beautifully” (Outcome Expectations). She wanted to bring “the Christmassy feeling of this music through the quality of sound and expressive, not hurrying from pizzicato to bowings, making sure the F sharp in the second chord sounds well, as it is usually covered by the piano” (Outcome Expectations).

For the second practicing session, she planned to “start with the easier parts” and then focus on some sort of “opposite interpretation with fingering changes, color changes” to get inspired by “new ideas” (Strategic Planning, Goal Orientation). Some notes from the previous session needed some “duration corrections” (Goal Setting). Xie also wanted to “improve pianissimo sounds while playing continuous vibrato without breaking the connection between long slurs and obtaining a beautiful legato without slowing down” (Outcome Expectations). In the last practicing session, Xie decided to “set an alarm to accomplish” what she wanted to (Strategic Planning) and to give herself “plenty of time to research and read about this music and the composers, as I am curious between keys and harmony in connection to scales” (Strategic Planning; Goal Orientation). She wanted to “focus on the big picture and how it transfers to different emotions” (Goal Orientation), and she also worked on how to “solve that quarter notes aren’t too long [by] playing scales and feeling the gaps of the notes with scales using the Bylsma technique” (Strategic Planning, Goal Orientation).

#### 5.3.2 Performance phase

During the first practice session, Xie worked to “find more interesting spots, and things that are repeated several times, so I use different keys to make it more interesting for me, otherwise I get bored” (Task Strategies). She approached both composers’ music using “the metronome for tempo, but thinking in bigger interconnected beats, because in the concert and rehearsals with the pianist, he will do that for me” (Help-Seeking). She also tried “different sounds, doing exaggerations and funny things, and listening to other Martucci works” (Task Strategies). Xie focused on her body, “trying to be relaxed during the shifts, and be confident mentally, trusting myself” (Task Strategies; Attention Focusing). She did try “different options for fingerings and bowings, [leaving] a few options open for the concert, so that it is not fully controlled, allowing for more improvised and fresh feeling during the performance” (Task Strategies). She was generally enjoying her practice as “I love this music and it is mostly expressive” (Self-Instruction; Interest Incentives). Overall, she realized that she needed to “practice with the pianist too, whatever I practice, it needs the bigger context, and to discuss ideas with him, to inform me musically and give me inspiration, I am curious” (Help-Seeking).

Although Xie was “stressed at the end [of the second practice session] due to time constrains” (Time Management), she engaged with “practicing the emotions, while being calm, chill, and focused” (Attention Focusing). She did all “I had planned, providing self-feedback, doing harmonic singing while playing the pizzicati, and separating hands for voice clarity” (Task Strategies). She thought that she needed to be “more structured and strategical when I practice this music again,” and she also experienced a certain “self-talk regarding curious musical trials” (Self-Instruction). The past practice session included the alarm set again, which made her feel “nervous.” Xie wanted to be more “aware of harmonic details,” planning where “to better jump and release to make the music more joyful” (Task Strategies). She was determined to draw connections between the “darkness and heavier f sharp minor scale [that brings me] a feeling of being a performing opera singer” (Imagery). She also worked on the “cello’s resonance in the lower register, trying different bow pressures on the string,” and thinking of coordinating this “with the pianist, for a clearer articulation and resonance balance” (Task Strategies). Across this last practice session, Xie had a kind of “revelation that there is the same rhythm across pieces (motif) which both composers use in my program. I should have filled in the gap between scales earlier, so good that I noticed this now” (Metacognitive Monitoring).

#### 5.3.3 Self-reflection phase

After every practicing session, Xie noticed a “rather steady and slight improvement in concentration” (Self-Evaluation) and that the selected passages “became better than the ones I did not work with the [OMMP] tool” (Casual Attribution). Overall, she felt that “practices and interviews surprisingly reduced a great amount of stage anxiety and stress before the concert” (Casual Attribution) and that she was “quite relaxed and calm [as] for once, it was a truly wonderful experience that I would never forget—all thanks to those reflections and focused practice” (Casual Attribution; Self-Satisfaction). After the concert, she said that she was “quite happy with the overall result, relaxed, and calm” and that most passages she had practiced “worked quite well” (Self-Satisfaction; Self-Evaluation). Even her friends told her that “it was a great performance and at the same time very me,” which made her feel content (Self-Satisfaction). Only minor things disturbed her during the concert, as there “were a few pizzicato places I was maybe trying too hard to bring out the harmony and then I pressed the strings too much and it did not end up sounding exactly like how I wanted [and] the beginning of Smyth sonata in the 3^rd^ movement that was slightly out of tune” (Casual Attribution). Yet, she realized that “no one was actually paying attention to the mistakes I made during the concert, instead the messages I was trying to deliver were being received successfully so I am very happy about it!” (Self-Satisfaction).

### 5.4 William

#### 5.4.1 Forethought phase

William chose the second Allegretto and the theme and variations of the final movement (Allegro) from Heinrich von Herzogenberg’s *Sonata for Piano and Cello no. 1 in A minor, op*. 52 (1886). He spent 60, then 49 and then 52 min for each of the three sessions, without breaks, to work on these passages. William said that during the pilot concert, he had experienced a “short of nervousness, which is very welcomed in the sense that the next performance will not be the first one anymore” (Goal Orientation). He thought that the selected passages were “a bit underprepared” (Self-Efficacy) as he “had the feeling my musical ideas were not too strong [and that] my phrasing had the opposite effect or not what I tried to do” (Task Interest/Valuing). William acknowledged he did “not know what to do with Herzogenberg’s musical material” (Goal Orientation), so he agreed to work on “issues of timing,” that the “harmony understanding should not be based on intuition, but on studying the score, that should inform me too” (Strategic Planning), and that “videoing the practicing both with the cello and the piano is priceless, plus lots of singing, as I intend to basically catch the idea from all directions” (Strategic Planning). This would help in “figuring out how to convey my musical ideas for these passages” (Strategic Planning).

The most important goal for William before starting practicing was to “find my own musical voice and not the standard around me, to open up perspectives and ideas and not to have a magical solution, [and to be] less stuck in checking the cello part [and instead] find the freedom by seeing the bigger picture” (Goal Orientation, Outcome Expectations). For this, he decided that the best strategies would be to alternate playing both the cello and piano parts, as well as analyzing the score and singing the music it aloud (Strategic Planning). He also thought it would be good to make “differences in musical surprises when material is repeated across the sections and building it faster until I am pleased with the clarity of nuances” (Strategic Planning). That kind of approach permeated all practicing sessions, but his goals and strategies became more focused toward “harmonic-based phrasing, finding character connections between movements at the emotional level” (Strategic Planning). He also planned to invest some time in “developing overall playing familiarity and reflecting and comparing the growth since I started” (Outcome Expectations). Other strategies he though relevant in the last session included “vibrato exercises, shifts, double stops, and thirds in the upper positions [as well as] deciding on the final fingerings and bowings” (Strategic Planning). William’s confidence across sessions regarding these goals and strategies grew progressively (Self-Efficacy), and he wanted to work in that way to “enjoy the process, for my own learning, and to contribute to the research project” (Goal Orientation).

#### 5.4.2 Performance phase

During the first practice session, William “shifted from the piano to the cello, to reading and singing the score” (Task Strategies), something he considered brings “much more awareness of all parts, something [they do not really] teach us at the university.” He also “mark[ed] a lot on the score, which I do not do a lot, but helped me musically” (Task Strategies), for instance “[jotting down] the harmonic reduction on the cello score to follow the chord changes” helped Willian “realize that the F sharp in the 5^th^ bar is something harmonically unexpected” or “find the magical moments when there is an odd sequence of chords” (Task Strategies, Imagery). He started making some decisions regarding bowings “in connection to [this passages’] singing qualities and harmony” (Task Strategies), and carefully studied “the headpins, and what the emotional idea of them is” (Imagery). During the second session, William felt “[in a] hurry and busy, but enjoying when feeling the interesting music by Herzogenberg, [with a certain] controversy of feeling satisfied to have time to do this [kind of practice]” (Self-Instruction, Interest Incentives). He started “building connection of phrases to be improved from the last session” (Task Strategies), working with a better focus “on the goals, the melody line of the piano, more singing, playing through” (Task Strategies), and mentioned that “the score analysis [from the previous session] revealed certain natural dramatic emotions” (Task Strategies, Imagery). William also started “waving hands to relax” while seeing the difference between “thought processes versus embodiment” (Attention Focusing). The last session involved a “good physical feeling, [an] enjoyment of flow, [and a] feeling of complete accomplishment for having done a good work, being on top of things” (Self-Instruction, Interest Incentives). He engaged with “warming up, vibrato exercises, shifts and extensions, strings crossing” (Task Strategies), and at the end he “wrote down the final fingerings and bowings” and sang the entire piece “to get into the music mood one last time” (Task Strategies).

#### 5.4.3 Self-reflection phase

After the concert, William realized that “video recording [the practicing sessions] was useful to avoid improvising during the concert” (Casual Attribution) and that “setting goals and strategies in advance and setting time aside to work on the chosen passage helped me focus while practicing” (Self-Evaluation; Casual Attribution). He also acknowledged that “setting myself goals other than instrumentally mastering the passage (harmonic and structural analysis) helped me in developing a very clear thought in how I want to play” (Casual Attribution). He felt confident about “all strategies in all sessions” (Self-Evaluation) and that the “selected passage worked out quite nicely [achieving] a slightly more refined performance and a feeling of sureness while playing” (Self-Evaluation; Affect). Overall, his “musical shape for the passage was quite successful and it had a natural flow” (Self-Evaluation); in fact, he “remember[ed] nothing whatsoever of playing the passage which might be a sign that I was into playing at that moment instead of being very analytical about my thoughts” (Self-Satisfaction). William also reported that there was “some room for improvement in how to balance things together with the piano, as [for instance,] I was forced to go slightly outside the boundaries of tone color suitable for the passage to stay balanced volume-wise” (Adaptive). All in all, he considered the intervention “an interesting experiment,” and that for further development, he could “have added some time rehearsing the passage together with my chamber music partner by using the [OMMP] tool” (Adaptive).

## 6 Discussion

A major imperative for any aspiring musician involves transitioning from a highly structured and organized learning environment under the external influence of a master teacher to a professional environment where the musician is placed under constant pressure to find solutions and prepare for performances mainly on their own. To successfully navigate this transition, musicians not only need to have developed the confidence and skills needed to survive as professional musicians (in line with López-Íñiguez and Bennett, 2020), but the abilities also to self-regulate their own learning in ways that allow them to take personal responsibility for monitoring and controlling their own performance (McPherson, 2022).

As described earlier in this article, the behavioral, cognitive, and affective sub-process described in the Self-Regulated Learning approach are now being used to develop microanalysis protocols that can effectively cue and focus the attention of learners before, during and after they have practiced or performed. A prime aim of these protocols is to help developing musicians maximize their ability to achieve at an even higher level. In this study, we explored the use of the Optimal Music Microanalysis Protocol (McPherson et al., 2015; Osborne et al., 2021) to examine (1) the types of self-regulated learning sub-processes musicians described to evaluate the quality of their individual practice session (and how these might be different across the four musicians), and (2) the ways in which the musicians reported the effectiveness of the microanalysis technique for improving their practice and subsequent performance.

For the results reported here, we can observe that the four musicians displayed varying degrees of ability to optimize their own performance as they monitored and controlled what they did, thought, and felt during each practice session. For example, Olga was constantly focusing on her negative feelings regarding her lack of strategies, and her thoughts and actions during practice did not match her goal of trying to convey the beauty of the phrasing in the repertoire she was mastering to her audience. Importantly, she directed most of her attention to technical aspects as she tried to achieve a better sound and intonation through repetitions of the selected passages. Lukas—who, like Olga, had short study sessions, kept working on similar goals, and was externally motivated to practice—spent part of his practicing focusing on shifting his ‘mood’ toward a more positive one, through body relaxation. He mostly wanted to achieve a better dynamic range to balance the excessive piano coverage, for which he worked on some sudden volume changes, playing slowly, created rhythmic variations of the passages that were softer, and achieving more clarity in the intonation by using the tuning machine.

Unlike Olga and Lukas, Xie set herself different and more complex goals and strategies for each subsequent practice session, and the amount of time spent was longer, although she also wanted to ensure that her body was relaxed and that she felt comfortable while performing. Her ability to plan strategically in different ways for each session means that she was able to focus her attention on “small details, thinking about the image of the passages, and not so much on the technique.” She set alarms for her practice, read books to inform her practice, used the metronome, video recorded, and critically analyzed her practice, used creative strategies for intonation, rhythms, and shifts. These tasks were oriented toward achieving the bigger picture and the musical expression of her selected passages, with an overall goal of enjoying learning for herself. William—who shared with Xie this internal process of learning and personal development—was even more fastidious with his practice, and besides doing many of the things his colleagues engaged with, he added singing, harmony reductions and analysis, and playing not only on the cello but also on the piano, which ensured that he knew his selected passages inside out. William not only wanted to make the music material more expressive, but to have a personal artistic voice that would stand out.

In general, all participants acknowledged that the OMMP helped them to achieve a more concentrated focus during their practice, and that they generally played the selected passages much better during the public concert than during the pilot performance. This was explained not only in terms of having had several additional weeks to practice, but to having had the possibility to reflect in a “deeper way than usual” [Olga], and to recognize that “without the [OMMP] tool, I definitely would have not reached this performance level for these passages” [Xie]. The OMMP therefore allowed each individual musician “the time to pause for a moment and think what I really want to achieve and assess whether I am actually achieving it” [William]. It also allowed them to “focus for once on one thing at the time” [Lukas].

We acknowledge that, even if the OMMP tool helped these four highly proficient musicians in enhancing their performances, there were striking differences in the goals and strategies that they chose for their practicing sessions. We explain this in terms of the presence or lack thereof of a *learner identity* in participants at the level of practice (i.e., Coll and Falsafi, 2010; Falsafi, 2011). A learner identity in musicians implies seeking to learn as a means of achieving a fulfillment of the self (e.g., Valdés et al., 2016) and acknowledging that “learning how to learn requires learning to be a learner” (Sinha, 1999, p. 41). This type of identity requires critically assessing “what we are not” (Reay, 2010, p. 2) with humility, for which recognizing ourselves as learners across the lifespan, undoubtedly sets musicians in an ideal learning zone and mindset that makes them more strategic and curious (e.g., López-Íñiguez and Bennett, 2021). Pairing this type of identity and approach to learning with the OMMP tool is an efficient way for musicians to get the most out of their practicing sessions and achieve creative artistic outputs during their concerts.

In conclusion, this article has outlined a technique for clarifying those sub-processes of a musician’s learning profile that would benefit from more purposeful attention before, during, and after practice sessions and public performances. Optimizing performance might involve devising strategies for encouraging musicians to be more attentive to the need to set goals and to identify ways of planning before practice has begun, in addition to working to motivate oneself, so that practice is even more efficient. In this regard, we agree with McPherson et al. (2019) and Osborne et al. (2021) who suggest that the technique outlined here provides a framework that can be adapted and modified to fit various learning contexts, depending on the developmental trajectory of the music learner.

## Data availability statement

The datasets presented in this article are not readily available because the datasets for this study are not available in order to ensure the anonymity of participants. Requests to access the datasets should be directed to guadalupe.lopez.iniguez@uniarts.fi.

## Ethics statement

The studies involving humans were reviewed by Uniarts Helsinki Research Ethics Committee. The studies were conducted in accordance with the local legislation and institutional requirements. The participants provided their written informed consent to participate in this study. Written informed consent was obtained from the individual(s) for the publication of any potentially identifiable images or data included in this article.

## Author contributions

GL-Í: Conceptualization, Data curation, Formal analysis, Funding acquisition, Investigation, Methodology, Project administration, Writing – original draft, Writing – review & editing. GM: Conceptualization, Resources, Validation, Writing – original draft, Writing – review & editing.
