# Supplementary material for: Using a music microanalysis protocol to enhance instrumental practice
Source: Front Psychol. 2024 Apr 2;15:1368074. doi: 10.3389/fpsyg.2024.1368074 (PMC11020086; doi:10.3389/fpsyg.2024.1368074)
Supplement: Supplementary file 1 [file Data_Sheet_1.PDF]

Name Date	BEFORE STARTING MY PRACTICE
	**The pieces I'm working on in this session are:**
**GOAL SETTING**<br>Describe the goals you have set for this practice session. These should ***not*** be written in general terms (e.g., "work on technique"). Be as specific as you can (e.g., *lighter touch left hand in bars 8-12/section B; effectively play through an 8-bar section of a new piece of music that has been identified as important*).	**The goals I want to achieve in this session are:**<br>1.____________________<br>2.____________________<br>3.____________________
**STRATEGIC PLANNING**<br>Explain the strategies that you believe will be critical for optimizing your performance.<br>1. What do you need to do to accomplish these goals?<br>*2.* Do you have any particular plans for how to learn/perform this piece/section/passage? *(e.g., using a strategy of first slowing the tempo, then gradually increasing it as performance is accomplished).*	**The strategies I plan to use to achieve these goals are:**<br>1.____________________<br>2.____________________<br>3.____________________
**SELF-EFFICACY**<br>Self-efficacy relates to your level of confidence that you *CAN* do something.<br>If you are completely confident that you can be successful in mastering your repertoire in this practice session then circle 100%. If you have no confidence that you can master your practice repertoire in this practice session then circle 0%. If your confidence lies somewhere in between, then circle the percentage that matches your confidence.	**My confidence that I CAN master the repertoire I practice in this session is:**<br>No Confidence — Complete Confidence<br>0% 10% 20% 30% 40% 50% 60% 70% 80% 90% 100%
**OUTCOME EXPECTANCIES**<br>How likely is it that you will successfully master your repertoire by the end of semester performance examination?	**My prediction that I WILL master my repertoire by the examination is:**<br>Unlikely — Highly Likely<br>0% 10% 20% 30% 40% 50% 60% 70% 80% 90% 100%

Osborne, M. S., McPherson, G. E., Miksza, P., & Evans, P. (2020). Using a microanalysis intervention to examine shifts in musicians' self-regulated learning. *Psychology of Music, 0*(0). https://doi.org/10.1177/0305735620915265

TASK INTEREST<br>How much do you like the repertoire you are practising in this session?	**My personal interest in the repertoire I am about to practise is:**<br>Not at all Interested ... Passionately Interested<br>0 1 2 3 4 5 6 7 8 9 10
TASK VALUE<br>How relevant is this repertoire to your personal long-term goals as a musician?	**The longer term value I place on the repertoire I am practising today is:**<br>Not at all Relevant ... Highly Relevant<br>0 1 2 3 4 5 6 7 8 9 10
GOAL ORIENTATION<br>What is the main purpose of this practise session?	**I'm practising today because** *(circle one that most applies for this practice session)*<br>I don't want to forget what I've learned<br>I want to play better than my peers<br>I'll let down my teacher if I don't<br>I want to achieve my personal best
SELF-CONTROL<br>Explain the ways you maintained your concentration and interest whilst using your planned strategies.<br>Concentration can include:<br>- specific tactics you used that related to the piece you were practicing<br>- instructions you gave yourself about the piece as you were practicing it<br>- how you organised the information in your mind<br>- how you planned your time during practice<br>- how you structured or modified your practice environment to get better results<br>if you asked for help, and if so, who/what did you consult?	DURING MY PRACTICE<br>**I focused my concentration by:**<br>____________________<br>____________________<br>____________________<br>____________________<br>____________________<br>____________________<br>____________________

Osborne, M. S., McPherson, G. E., Miksza, P., & Evans, P. (2020). Using a microanalysis intervention to examine shifts in musicians' self-regulated learning. *Psychology of Music, 0*(0). https://doi.org/10.1177/0305735620915265

Interest can include:<br>- things you said to yourself to remind yourself of the goals e.g., praising and/or rewarding yourself<br>- trying out different ways of expressing the music	**I maintained my interest by:**
SELF-OBSERVATION<br>What did you do to judge how effective your practice strategies were?<br>This can include:<br>- your self-talk, the things you were saying to yourself as you were working through the piece and problem solving<br>- ways you kept a record of your progress (e.g., using your phone to record your playing)	**I monitored my practice by:**

-

Osborne, M. S., McPherson, G. E., Miksza, P., & Evans, P. (2020). Using a microanalysis intervention to examine shifts in musicians' self-regulated learning. *Psychology of Music, 0*(0). https://doi.org/10.1177/0305735620915265

SELF-EVALUATION
How did you go in this practice session? Evaluate how productive and effective your strategies were,
e.g., *I didn't practice effectively today because the strategies I identified weren't appropriate*
e.g., *learning that 8-bar section was easy today; perhaps next time I can be more ambitious about what I fit into a practice session*

AFTER MY PRACTICE WAS COMPLETED

My practice was *(circle the number that most applies)*

Not at all Effective ______ Highly Effective

0 1 2 3 4 5 6 7 8 9 10

Why? ______________________________

____

____

Describe the practice session by rating the following words:

Different ______ Typical

0 1 2 3 4 5 6 7 8 9 10

Unfocused ______ Focused

0 1 2 3 4 5 6 7 8 9 10

Stimulating ______ Tedious

0 1 2 3 4 5 6 7 8 9 10

Frustrating ______ Satisfying

0 1 2 3 4 5 6 7 8 9 10

The strategies I chose to achieve my goals were:

Not at all Effective ______ Highly Effective

0 1 2 3 4 5 6 7 8 9 10

Osborne, M. S., McPherson, G. E., Miksza, P., & Evans, P. (2020). Using a microanalysis intervention to examine shifts in musicians' self-regulated learning. *Psychology of Music, 0*(0). https://doi.org/10.1177/0305735620915265

CAUSAL ATTRIBUTION

Assess your practice based on the goals and criteria you set yourself before you started this practice session.

Was this because of *(circle Yes or No):*

Poor effort and work ethic	Yes	No
Lack of ability to master repertoire	Yes	No
Having a bad day	Yes	No
Lack of help from my teacher	Yes	No
Lack of planning	Yes	No
Lack of focus	Yes	No
Low energy (tired/lethargic/sick)	Yes	No
Too many distractions	Yes	No
Unfixable errors	Yes	No
Not enough time	Yes	No
Not achieving personal goals	Yes	No

SELF-SATISFACTION/AFFECT

Explain the reasons you feel your practice was satisfying or disappointing.

My practice session was:

Completely Disappointing — Completely Satisfying

0 1 2 3 4 5 6 7 8 9 10

ADAPTIVE/DEFENSIVE

How keen are you to get to your next practice session?

Describe your feelings and emotional reactions to your judgements about how well you went in this practice session.

. . . and this caused me to feel *(circle those that apply)*

Empowered	Enthusiastic	Interested
Focused	Planned	Excited
Normal	Disinterested	Unmotivated
Helpless	Eager	Determined

My overall estimate of the percentage of this practice session that was focused and concentrated is:

0% 10% 20% 30% 40% 50% 60% 70% 80% 90% 100%

For this session I practised for: _________ (mins)

Osborne, M. S., McPherson, G. E., Miksza, P., & Evans, P. (2020). Using a microanalysis intervention to examine shifts in musicians' self-regulated learning. *Psychology of Music, 0*(0). https://doi.org/10.1177/0305735620915265
